# Cardenolides in the defensive fluid of adult large milkweed bugs have differential potency on vertebrate and invertebrate predator Na^+^/K^+^-ATPases

**DOI:** 10.1098/rsos.231735

**Published:** 2024-06-05

**Authors:** P. Rubiano-Buitrago, S. Pradhan, A. A. Aceves, S. Mohammadi, C. Paetz, H. M. Rowland

**Affiliations:** ^1^ Research Group Predators and Toxic Prey, Max Planck Institute for Chemical Ecology, Jena, Germany; ^2^ Research Group Biosynthesis/NMR, Max Planck Institute for Chemical Ecology, Jena, Germany

**Keywords:** *Oncopeltus fasciatus*, cardiac glycoside, black-headed grosbeak, toxin–receptor interaction, predator–prey

## Abstract

Aposematic animals rely on diverse secondary metabolites for defence. Various hypotheses, such as competition, life history and multifunctionality, have been posited to explain defence variability and diversity. We investigate the compound selectivity hypothesis using large milkweed bugs, *Oncopeltus fasciatus*, to determine if distinct cardenolides vary in toxicity to different predators. We quantify cardenolides in the bug’s defensive secretions and body tissues and test the individual compounds against predator target sites, the Na^+^/K^+^-ATPases, that are predicted to differ in sensitivity. Frugoside, gofruside, glucopyranosyl frugoside and glucopyranosyl gofruside were the dominant cardenolides in the body tissues of the insects, whereas the two monoglycosidic cardenolides—frugoside and gofruside—were the most abundant in the defensive fluid. These monoglycosidic cardenolides were highly toxic (IC_50_ < 1 μM) to an invertebrate and a sensitive vertebrate enzyme, in comparison to the glucosylated compounds. Gofruside was the weakest inhibitor for a putatively resistant vertebrate predator. Glucopyranosyl calotropin, found in only 60% of bugs, was also an effective inhibitor of sensitive vertebrate enzymes. Our results suggest that the compounds sequestered by *O. fasciatus* probably provide consistency in protection against a range of predators and underscore the need to consider predator communities in prey defence evolution.

## 1. Introduction

Toxins and colourful warning signals characterize the defences used by aposematic species against predators [1,2]. Aposematic animals acquire toxins by sequestering plant-specialized metabolites [3] or by de novo synthesis [4,5]. In prey that sequester toxins, variability in the quantity and biochemical profile of chemical defences is common, both within and between species (e.g. in poison frogs, *Dendrobates tinctorius* [6], *Heliconius* butterflies [7,8], ladybirds [9] and nudibranchs [10]). Providing and evaluating evolutionary explanations for this variation, as well as alternative explanations, can shed light on the ecological relevance of chemical defence composition and how natural selection constrains or promotes toxin diversity [2].

There are a number of explanations for the variability and complexity of defensive chemicals, including the stochastic nature of the environments within which prey organisms exist and develop, selective sequestration of compounds, life-history effects on defences and variation in the selection pressures exerted by predators and other natural enemies [2]. For example, the concentration of sequestered cardenolides in monarch butterflies (*Danaus plexippus*) varies depending on host plant chemistry [11,12], and host plant chemistry is impacted by environmental conditions [13]. Chemical diversity can also be explained by selective sequestration [14–16]. Large milkweed bugs (*Oncopeltus fasciatus*) sequester intermediate and more polar cardenolides from milkweeds (Apocynaceae: Asclepiadoideae) and frequently diverge from those of their host plants even if the host plants have distinct chemical profiles [17,18]. For example, *O. fasciatus*, when fed on seeds of the tropical milkweed, *Asclepias curassavica*, sequesters higher concentrations of the cardenolides frugoside and gofruside than are available in the seeds [19]. The greater sequestration of these compounds is not explained by their effect on the biological activity on the bug’s target site, because the two compounds differ in potency—frugoside is the most inhibitory, whereas gofruside is among the weakest inhibitors [19].

An alternative hypothesis for the bug’s sequestration pattern is that, just as individual plant compounds are targeted at distinct herbivores (i.e. compound selectivity hypothesis [20,21]), the milkweed bug’s chemical profiles might be explained by the toxicity of these chemical defences to different natural enemies [2,22–24]. Milkweed bugs are considered partial migrants [25], which could result in their exposure to a diversity of natural enemies. For example, in another milkweed herbivore, the oleander aphids, *Aphis nerii*, Malcolm [26] identified nine predator species that varied in their ability to exploit *A. nerii* as a food source. An alternative approach for exploring the potential specificity of sequestered chemical defences on predators is to test isolated compounds *in vitro* [27]. This has proved successful in other systems: monarch butterflies sequester cardenolides from milkweed leaves that are less potent against their own target site than the dominant cardenolides available in the leaves, but the sequestered cardenolides retain high potency against the target sites of sensitive vertebrate target sites [27].

Here, we build on the methods of Agrawal *et al.* [27] and identify and quantify the cardenolides sequestered by individual large milkweed bug adults reared in the laboratory on tropical milkweed seeds (*A. curassavica*). *Asclepias curassavica* is present in the migration range of milkweed bugs [28,29]. We combine this quantification with tests of the inhibitory capacity of the main compounds sequestered by the bugs on the Na^+^/K^+^-ATPases of three predator species that we use as a proxy for natural enemy diversity: the black-headed grosbeak (*Pheucticus melanocephalus*), which feeds on thousands of cardenolide-sequestering monarch butterflies in their large overwintering aggregations in Mexico [30,31] and has evolved amino acid substitutions in the Na^+^/K^+^-ATPases, which may confer target site insensitivity [32]; the zebra finch (*Taeniopygia castanotis*), as a comparative passerine which does not have any putatively resistance-conferring amino acid substitutions in the Na^+^/K^+^-ATPase (gene ID 100190719); and the giant Asian mantid (*Hierodula membranacea*) because mantids have been repeatedly used as predators in experiments with milkweed bugs and monarch butterflies [33–35] and because other species of mantid (e.g. *Tenodera sinensis*) have been reported to vomit or regurgitate after eating milkweed bugs [33,36]. We used the porcine Na^+^/K^+^-ATPase enzyme as reference following Agrawal *et al.* [27].

We show that cardenolides sequestered into the defensive fluids of milkweed bugs are dominated by two monoglycosidic cardenolides—frugoside and gofruside—and that these compounds have differential effects on the target sites of birds and invertebrate predators. We advance the compound selectivity hypothesis, suggesting that specialist herbivores sequester compounds that are toxic to a range of potential enemies. Support for this hypothesis provides an explanation for the diversity of cardenolides found in large milkweed bugs.

## 2. Material and methods

### 2.1. Study species

*Oncopeltus fasciatus* were obtained from a long-term laboratory colony at the University of Giessen in 2019. This colony originates from the United States and was acquired by the University of Hamburg in 2015. We reared the bugs on organic sunflower seeds (Alnatura, Darmstadt, Germany) in terrarium boxes (37 × 22 × 25 cm) lined with tissue paper and provided them with ad libitum water in Eppendorf tubes plugged with dental cotton. The boxes were equipped with pieces of cotton wool for oviposition. The colonies were maintained in an incubator at 28°C and 70% humidity with an 18 L : 6 D cycle and a temperature of 18°C at night (Polyklima PK 520-LED).

*Asclepias curassavica* seeds were obtained from Jelitto Perennial Seeds (Schwarmstedt, Germany).

*Hierodula membranacea* were obtained from M&M Wust—Mantids and More (Muhlheim am Main, Germany). Twenty-five individuals were obtained at the L4 stage and reared in individually double-ventilated boxes (19 × 19 × 19 cm). We provided them with greenbottle fly pupae as a food source, twice per week (two pupae during the nymph stages and three when they reached the adult stage). Mantids were sprayed with water every 2 days for hydration. The mantids were kept in an incubator (Snijders Scientific premium, Tilburg, The Netherlands, with an Imago 500 JUMO controller, Fulda, Germany) at 28°C and 70% humidity with an 18 L : 6 D cycle and a temperature of 18°C at night.

*Taeniopygia castanotis* (zebra finch) brain tissues were obtained from a breeding colony at the University of St Andrews, UK, under Home Office licence 70/8159. Individual brains were dissected and flash-frozen on dry ice before being shipped from St Andrews to the Max Planck Institute for Chemical Ecology, Jena, Germany, where they were kept at −80°C until used in the assays.

*Pheucticus melanocephalus* (black-headed grosbeak) ATPA1 and ATPB1 genes were synthesised (GeneArt; Invitrogen), codon optimised for *Spodoptera frugiperda* and cloned by GeneArt (Invitrogen) in pFastBac Dual plasmid with ATP1B1 under p10 promoter and ATP1A1 under P_PH_ promoter. Final plasmids were verified by sequencing (accession number 196465).

### 2.2. Sequestration behaviour, collection of dorsolateral space fluid and extraction of cardenolides from whole bodies

For the purpose of this experiment, we established two distinct colonies of milkweed bugs by randomly selecting individuals from our existing stock colonies during standard insect husbandry and colony management. We selected 20 mating pairs and an additional 12 adults and 12 L5 larvae from the stock colonies that had been reared on sunflower seeds. The new colonies were provided with *A. curassavica* seeds ad libitum. These colonies and their resulting offspring were maintained on the same batch of milkweed seeds for five generations. Seeds and water were replenished bi-weekly and weekly, respectively.

The large milkweed bug has evolved a vacuolated double-layered integument or dorsolateral space (DLS) where it accumulates the cardenolides sequestered from seeds. Upon mechanical stress, adult bugs release a complex mixture of cardenolide-rich fluid from exit points in the thin cuticle of the DLS [37,38]. After release, the fluid is held in droplets. This increases the likelihood that predators come into contact with the fluid while subjugating prey. To collect the defensive fluid, we randomly selected 66 adults (33 males and 33 females, without controlling for reproductive stage), from the fifth generation of each colony. The adults were weighed, and then P.R.-B. manually stressed them by squeezing between forceps to elicit the release of the defensive fluids [38]. P.R.-B. collected the defensive fluids from each individual separately, in disposable 1–5 μl micropipettes made of Duran glass with a ringmark (Hirschmann Laborgeräte GmbH, Eberstadt, Germany). The micropipettes with the fluid were washed thoroughly with 100 μl MeOH immediately after collection. The solvent was evaporated at ambient temperature under N_2_ gas. Later the fluid was resuspended in 50 μl of MeOH for liquid chromatography-mass spectrometry (LC–MS) measurements.

After manual stress, bugs were weighed again, frozen at −80°C, and then freeze-dried overnight at −85°C and 0.014 mbar (Martin Christ Alpha 1–2 LD Freeze Dryer, Osterode am Harz, Germany). We followed the methods of Pokharel *et al*. [39] to extract the remaining cardenolides in the body and those not fully collected from the DLS. The freeze-dried tissues of the adults were placed into a FastPrep matrix tube (MP Biomedicals Germany GmbH, Eschwege, Germany) with approximately 450 mg of 2.3 mm zirconium/glass pellets (Carl Roth GmbH + Co. KG, Karlsruhe, Germany) and 1 ml of MeOH (Rotisolv 99.9%, Carl Roth GmbH, Karlsruhe, Germany). The sample was homogenized in the FastPrep 24–5G Tissue Homogeniser (MP Biomedicals Germany GmbH, Eschwege, Germany) in two cycles of 45 s at 6.5 m s^-1^, with a pause time of 100 s between cycles. The homogenate was then centrifuged at 16 000 RCF for 3 min, and 700 μl of supernatant was collected. We repeated the homogenization with the addition of 1 ml of MeOH another two times. The three collected supernatants per bug were pooled and washed with MeOH through a Chromabond HR-X 86 μM cartridge 200 mg (Macherey-Nagel GmbH, Düren, Germany). The extracts obtained were dried under N_2_ gas and weighed. They were then diluted in 200 μl of MeOH for the LC–MS analysis.

### 2.3. Liquid chromatography-mass spectrometry spectrometric quantification of *Asclepias* cardenolides in dorsolateral space fluid and bodies

We analysed the concentration of eight cardenolides in the DLS fluid and dried tissues of *O. fasciatus* using a linear calibration method for high-performance liquid chromatography coupled to high-resolution mass spectrometry (HPLC–HRMS; see Rubiano-Buitrago *et al.* [40]. We used cardenolide standards derived from our previous isolations from *A. curassavica* seeds (see Rubiano-Buitrago *et al.* [40]). These standards were glucopyranosyl-12-β-hydroxyl coroglaucigenin, 16α-hydroxycalotropin, allopyranosyl coroglaucigenin, glucopyranosyl frugoside, glucopyranosyl gofruside, glucopyranosyl calotropin, frugoside and gofruside [40]. We injected 1 μl of the resuspended DLS fluid and 4 μl of the dried tissue extracts of *O. fasciatus* into the HPLC–HRMS and followed the chromatography conditions and quantification parameters described by Rubiano-Buitrago *et al.* (2023) (see also the electronic supplementary material, method S1) [40].

### 2.4. Functional Na^+^/K^+^-ATPase assays

Preparations of Na^+^/K^+^-ATPases were obtained by homogenization of dissected brains (*T. castanotis*, zebra finch, and *H. membranacea*, giant Asian mantid), from commercially obtained enzyme (*Sus scrofa domesticus*, domestic pig), and expression of ATP1A1 and ATP1B1 subunits of the Na^+^/K^+^-ATPase of the black-headed grosbeak in insect Sf9 cells (Invitrogen).

### 2.5. Preparation of lysates of zebra finch and giant Asian mantid

We sliced and weighed 5 mg from three different zebra finch brains and dissected the head capsule of five female giant Asian mantids to obtain the central body with intact optic lobes. Neural tissues were transferred to a 1 ml glass grinder (Wheaton Dounce tissue grinder, 1 ml, no. 357538) and homogenized in 500 μl distilled water. Zebra finch homogenates were transferred to a 50 ml Falcon tube on ice and resuspended with 15 ml resuspension buffer (0.25 M sucrose, 2 mM ethylenediaminetetraacetic acid and 25 mM HEPES/Tris; pH 7.0). Samples were sonicated at 85 W (Fisherbrand Model 120 Sonic Dismembrator, no. 12337338) for three 45 s intervals at 0°C, followed by centrifugation for 30 min at 10 000*g* (Sigma 3–18K, no. 10290) at 4°C to remove debris. The supernatant was collected and further centrifuged for 60 min at 100 000*g* at 4°C (Optima Max-XP tabletop ultracentrifuge, no. 393315) to isolate the membrane fraction. The pelleted membranes were washed twice and resuspended in 1 ml Milli-Q water (Milli-Q direct water purification system, Merck, no. C85358) and stored at −20°C. Giant Asian mantid homogenates were frozen to −80°C and then freeze-dried overnight and then resuspended in 1800 μl distilled water. Each sample was divided into aliquots of 600 μl and sonicated twice in an ice water bath (Bandelin Sonorex, no. Z659584) for 5 min. Samples were centrifuged at 3000 r.p.m. for 5 min and used to perform functional assays.

### 2.6. Expression of black-headed grosbeak Na^+^/K^+^-ATPase

Recombinant Na^+^/K^+^-ATPases were expressed after infection of Sf9 cells with P0 virus stock following the optimized baculovirus expression system described by Scholz and Suppmann [41]. The cells were pelleted by centrifugation, resuspended and sonicated to disrupt membranes and further centrifuged to remove cell debris. Cell membranes were pelleted by ultracentrifugation of the supernatant and finally resuspended in Milli-Q water (electronic supplementary material, method S2) [42]. Prior to the Na^+^/K^+^-ATPase inhibition assay, the black-headed grosbeak protein was verified by SDS–PAGE/Western blotting following the methods of Mohammadi *et al.* [43] (see the electronic supplementary material, figure S1) and quantified by ELISA (see the electronic supplementary material, method S2d).

### 2.7. Na^+^/K^+^-ATPase inhibition assay

The inhibitory effects of increasing concentrations of four cardenolides (ouabain, glucopyranosyl frugoside, frugoside and gofruside) on the black-headed grosbeak, zebra finch and giant Asian mantids were determined by photometric measurement of inorganic phosphate released from enzymatic ATP hydrolysis while subtracting the background ATPase activity following Petschenka *et al.* [44] (see the electronic supplementary material, method S2e). The assessment of the inhibitory impact of glucopyranosyl calotropin was limited to the black-headed grosbeak and zebra finch species. This stemmed from the restricted availability of the compound, which only permitted testing on two predator pumps. The inhibitory effects of the five compounds for the porcine ATPase were taken from Rubiano-Buitrago *et al.* [19] for comparison. All assays were run in three biological replicates, and the average of two technical replicates of each biological replicate was used for subsequent statistical analyses. Raw data are available in the data repository [45].

### 2.8. Data analysis

#### 2.8.1. Sequestration

To determine the percentage of cardenolide content measured by the available standards and the linear calibration method, we summed all areas in the MS trace that we recognized as cardenolides based on the fragmentation patterns and masses and calculated the percentage of the samples that were not represented by the eight known cardenolides (electronic supplementary material, table S1 and figure S2).

To determine the concentration of cardenolides in μg μl^-1^ of DLS fluid, we summed the concentrations of the eight cardenolides present in the DLS fluid and then divided this sum by the volume ascertained for each individual. To calculate the μg cardenolide mg^-1^ of dry weight of tissue, we divided the sum per dried weight [46]. To compare the amount of individual cardenolides in the DLS fluid and in the bodies of milkweed bugs, we first used Levene’s test to assess the homogeneity of variance within a tissue type. There was significant heterogeneity of variance between compounds for both the DLS fluid and the body tissues (*F*_7,475_ = 15.99, *p* < 0.0001; *F*_7, 520_ = 5.94, *p* < 0.0001, respectively). We analysed the difference between the cardenolides within the DLS fluid and within the body tissues using separate Welch’s ANOVA for unequal variances and compared the cardenolides to one another with a Games–Howell post hoc test for unequal variances.

#### 2.8.2. Na^+^/K^+^-ATPase inhibition

For cardenolide inhibition, we converted the calibrated absorbance values to the percentage of non-inhibited Na^+^/K^+^-ATPase activity based on measurements from the control wells. We fitted inhibition curves by nonlinear fitting using a four-parameter logistic curve, with the top and bottom asymptotes set to 100 and 0, respectively, using the nlsLM function of the minipack.lm library in R [47]. From this we calculated the half maximal inhibitory concentration (IC_50_) values for each biological replicate. We compared the log10 IC_50_ values of individual cardenolides for each Na^+^/K^+^-ATPase enzyme using a linear model, testing the interaction between predator enzyme and cardenolide, and compared the inhibitory capacity of each compound pairwise using Tukey’s post hoc test. We calculated the fold differences between the IC_50_ values of individual cardenolides for black-headed grosbeak, zebra finch and giant Asian mantid versus the IC_50_ values on the porcine enzyme.

All analyses were conducted in R (version 1.4.1717).

## 3. Results

### 3.1. Defensive secretion volume and sequestered cardenolides in *Oncopeltus fasciatus*

Adults of *O. fasciatus* released between 0.2 and 2.0 μl of fluid from the DLS after manual stress (mean ± s.e.: 0.98 ± 0.06 μl, *n* = 66). Heavier bugs secreted a significantly larger volume of DLS fluid than lighter bugs (*R* = 0.6, *p* < 0.0001; electronic supplementary material, figure S3). For the DLS fluid, the eight cardenolides that we quantified through linear calibration corresponded to 88.4 ± 0.53% of the total cardenolide area in the samples (electronic supplementary material, figure S2). For the extract of the bug’s tissue, the eight cardenolides represented 72.3 ± 0.7% of the cardenolide area in the LC–MS trace (electronic supplementary material, figure S2). Two undescribed cardenolides (compounds D and E) were consistently more abundant in the tissue samples compared with the DLS fluid (electronic supplementary material, figure S4).

**Figure 1.**
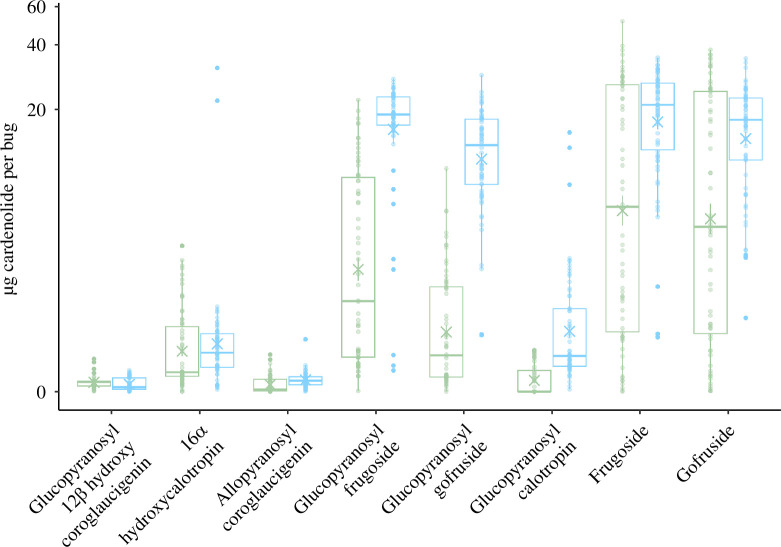
Cardenolides (µg per bug) in *O. fasciatus* adults in the DLS fluid (green) and in the body tissue after depletion of the fluid (blue; x indicates the mean, *n* = 66). Compounds are arranged from polar to non-polar based on HPLC retention times. Note that the *y*-axis is in pseudo-log scale.

There was a significant difference between the concentration of cardenolides in the dried tissues after depletion of the fluid (*F*_7,209.05_ = 223, *p* < 0.0001). Frugoside, gofruside and glucopyranosyl frugoside did not differ significantly in abundance (electronic supplementary material, table S3) and were significantly more abundant than the other cardenolides in the dried tissues (figure 1; electronic supplementary material, table S3). Glucopyranosyl gofruside was equally abundant as gofruside (estimate = 3.63, confidence interval (CI) = −0.08–7.35, *p* = 0.059) but was significantly less abundant than frugoside and glucopyranosyl frugoside (estimate = 6.86, CI = 2.91–10.8, *p* < 0.0001; estimate = −5.03, CI = −8.29 to −1.78, *p* = 0.0001).

We did not find glucopyranosyl calotropin in 60% of samples from bugs’ DLS fluid. There was a significant difference between the concentration of the eight cardenolides in the DLS fluid (*F*_7, 169.99_ = 43.176, *p* < 0.0001). Frugoside and gofruside did not differ significantly in abundance (estimate = −1.03, CI = −8.01–5.94, *p* > 0.05) and were significantly more abundant than the other cardenolides in the DLS fluid (figure 1; electronic supplementary material, table S4). Glucopyranosyl frugoside was significantly more abundant than glucopyranosyl gofruside and glucopyranosyl calotropin (estimate = −3.78, CI = −6.18 to −1.37, *p* = 0.0001; estimate = −4.88, CI = −7.17 to −2.60, *p* < 0.0001, respectively). Glucopyranosyl gofruside and glucopyranosyl calotropin had equally low abundance to 16α-hydroxycalotropin (estimate = 0.79, CI = −0.18–1.58, *p* = 0.23; estimate = −0.41, CI = −0.86–0.04, *p* = 0.11, respectively), though glucopyranosyl gofruside was more abundant than glucopyranosyl calotropin (estimate = −1.11, CI = −1.91 to −0.30, *p* = 0.001).

### 3.2. Cardenolide toxicity to predators tested by functional Na^+^/K^+^-ATPase assay

**Figure 2.**
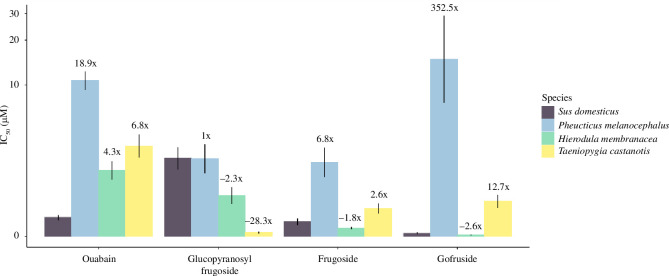
Mean molar concentration of sequestered milkweed toxin necessary to cause 50% inhibition of the predator Na^+^/K^+^-ATPase enzyme (IC_50_ ± s.e., *n* = 4–6 per compound). Higher values on the *y*-axis indicate that the enzyme is more resistant to the cardenolide. Note that the *y*-axis is in pseudo-log scale.

According to the criteria defined by Agrawal *et al.* [48], which classify molecules with IC_50_ > 100 µM as nontoxic, 10 to 100 µM as moderately toxic, IC_50_ < 10 µM as toxic and IC_50_ < 1 µM as highly toxic (also refer [49]), we found that the coroglaucigenin cardenolides (glucopyranosyl frugoside and frugoside) are toxic (IC_50_< 10 μM) for black-headed grosbeak and highly toxic (IC_50_< 1 μM) for zebra finch. For the invertebrate predator*,* the giant Asian mantid, the monoglycosidic cardenolides (frugoside and gofruside) were highly toxic (IC_50_ < 1 μM; figure 2; electronic supplementary material, table S5).

We found a significant interaction between cardenolide and predator Na^+^/K^+^-ATPase (*F*_9,70_ = 30.06, *p* < 0.0001) and therefore analysed how different species were affected by each cardenolide, splitting the dataset by compound. We found a significant difference between the species’ responses to ouabain (*F*_3,18_ = 41.76, *p* < 0.0001). Ouabain was 18.9 times less inhibitory for black-headed grosbeak than for pig (estimate 1.28, CI = 0.95–1.62, *p* < 0.0001), 4.3 times less inhibitory for the giant Asian mantid (estimate = 0.62, CI = 0.31–0.92, *p* =0.0001) and 6.8 times less inhibitory for the zebra finch enzyme (estimate = 0.81, CI = 0.51–1.12, *p* < 0.0001).

Glucopyranosyl frugoside had significantly different effects on the predator Na^+^/K^+^-ATPase (*F*_3,16_ = 40.52, *p* < 0.0001; electronic supplementary material, figure S7). It was 28.3 times more inhibitory to zebra finch than to pig (estimate = −1.46, CI = −1.88 to −1.04; *p* < 0.0001) and 2.3 times more inhibitory to the giant Asian mantid, though this was not significant at the alpha 0.05 level (estimate −0.38, CI = −0.80–0.04, *p* = 0.08). It was not significantly different in potency for black-headed grosbeak (estimate = −0.004, CI = −0.45–0.44, *p* = 0.99).

Frugoside had significantly different effects on the predators’ sodium pumps (*F*_3,18_ = 23.43, *p* < 0.0001). It was 6.8 times less inhibitory for black-headed grosbeak than for pig (estimate = 0.80, CI = 0.45–1.16, *p* < 0.0001) and 2.6 times less inhibitory for zebra finch, though this was not significant at the alpha 0.05 level (estimate = 0.30, CI = −0.04–0.64, *p* = 0.09). It was not significantly different in potency to the giant Asian mantid (estimate = −0.23, CI = −0.58–0.13, *p* = 0.31).

Gofruside also had significantly different effects on the pumps (*F*_3,18_= 48.80, *p* < 0.00001). It was 352.5 times less inhibitory for black-headed grosbeak than the pig (estimate = 2.30, CI = 1.62–2.97, *p* < 0.0001). Gofruside was 12.7 times less inhibitory to zebra finch than the pig (estimate = 1.15, CI = 0.50–1.79, *p* = 0.0005) and did not differ in inhibitory potential between the pig and the giant Asian mantid (estimate = −0.40, CI = −1.08–0.27, *p* = 0.36).

**Figure 3.**
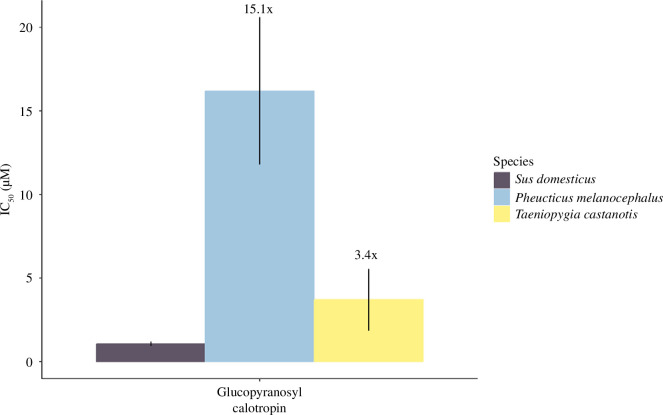
Mean molar concentration of glucopyranosyl calotropin necessary to cause 50% inhibition of the predators’ enzyme (IC_50_) shown as means ± s.e., *n* = 5. Higher values on the *y*-axis indicate that the enzyme is more tolerant to the cardenolide.

The IC_50_ values also differed significantly for glucopyranosyl calotropin (figure 3; *F*_2, 13_ = 10.55, *p* = 0.002). Glucopyranosyl calotropin was 15.1 times less inhibitory for black-headed grosbeak than for pig (estimate = 1.08, CI = 0.45–1.71, *p* = 0.002) and 3.4 times less inhibitory to zebra finch than the porcine enzyme, but this was not statistically significant at the alpha 0.05 level (estimate = 0.32, CI = −0.31–0.95, *p* = 0.41).

## 4. Discussion

We investigated the nature and function of chemical defence diversity in large milkweed bugs, testing the hypothesis that compound diversity represents differential toxicity to different natural enemies. We observed an order-of-magnitude difference in the variability of cardenolide concentrations in the defensive secretions and body tissues of milkweed bugs. This within-population variation in the quantity of secondary metabolites is a typical feature of aposematic animals [2] and can be maintained if predators are more willing to eat prey with a consistent level of defence, compared to prey with variability in their defences [50]. We also identified variability in the biochemical profile between individuals. Among the eight compounds that we quantified, which collectively constitute 88% of the cardenolides present in the defensive fluid, a specific compound (glucopyranosyl calotropin) was detectable only in a subset of individuals. Several hypotheses have been put forward to explain the variability observed in defensive profiles, including competition, life history and additional functions of defensive substances [2]. Our tests of the multiple enemy hypothesis involved measuring the inhibitory properties of a subset of the individual components against the target sites of several predators. We found that frugoside, which is one of the most abundant sequestered compounds in the defensive fluid, acts as a robust defence against invertebrate and sensitive vertebrate enzymes. Gofruside, another of the most abundant sequestered compounds in the defensive fluid, varied in enzyme inhibition among various predators, exerting its strongest inhibitory effects on an invertebrate predator, while demonstrating comparatively weaker inhibition towards the target site of the resistant predator. Glycosylated cardenolides are generally considered to be more toxic than corresponding genins in whole organism vertebrate assays [19], but our one comparison found the deglycosylated version of glucopyranosyl frugoside was more inhibitory for two out of four enzymes. While defence against predators is perhaps the function most often associated with sequestration and defensive secretions, our results add to the growing literature showing the ecological relevance of the chemical composition of sequestered defences [51–53] and reveal the evolutionary explanations for toxin diversity [2].

The total cardenolide concentration of the bugs measured in this study ranged from 25.4 to 208.7 μg per bug. This range is similar to that reported by Isman [18], who found that some milkweed bugs lack cardenolides (or have levels of cardenolides below the detectable limit of the measuring equipment), whereas others contain up to 375 μg. The variability in the bug’s sequestration behaviour in our study cannot alone be accounted for by the cardenolide content of the host plant because the bugs in our study were provided with an ad libitum supply of seeds of *A. curassavica*. This within population variation may reflect genetic differences in the individuals’ capacity to sequester [54], differences in individual physiological state [55,56] or the absence of predation pressure and the relaxed selection in the laboratory [53]. The higher variance in the concentration of cardenolides in the defensive fluid could also be owing to the fact that we were only able to collect a fraction of the DLS content during manual stress. Duffey and Scudder [14] suggested that only half of the DLS content is collectable or released upon manual stress and the total vacuolar volume is difficult to determine [14,37]. The concentration that we measured does, however, control for the volume collected. The variance could also reflect sex differences in sequestration, but we found no differences between male and female cardenolide concentrations in the DLS fluid (see the electronic supplementary material, S6 and also Moore and Scudder [17]). We did not control for insects’ reproductive stage, or age, which might contribute to variation [56]. Testing whether predators are able to detect the variation present in the chemical defence will be important for understanding if such variation is subject to differential selection and therefore how intraspecific variation in chemical defence concentration can be maintained [6].

The diversity of defence compounds that we report in the defensive secretion and bodies of large milkweed bugs is characteristic of many aposematic animals including poison frogs, *Lepidoptera* [51,57–59], nudibranchs [10,60], *Coleoptera* [61,62] and *Orthoptera* [63]. That specialist herbivores concentrate some toxins while not sequestering others has long been known [64,65]. A common question about defensive variability is whether it represents ‘ecological noise’, variation caused by the stochastic nature of prey environments, or is of no adaptive evolutionary significance [2,66]. We previously reported higher concentrations of the cardenolides frugoside and gofruside in the bugs than is available in the seeds on which they feed [19,40]. These two compounds have contrasting potency towards the *O. fasciatus* target site Na^+^/K^+^-ATPase: frugoside is the most inhibitory, whereas gofruside is among the weakest inhibitors. Our present results, which demonstrate that frugoside has similar levels of toxicity against both resistant and sensitive predator enzymes, could explain why the bugs sequester a more potent inhibitor specific to their own target site. Our results are consistent with those reported by Lawrence *et al.* [52], who found core alkaloids in poison frog defences that may provide the consistency in protection necessary for aposematic warning signals to be maintained. Gofruside, characterized as a modest inhibitor of the bug’s target site, is toxic to predators with sensitive target sites but is a weaker inhibitor of resistant enzymes. Understanding how *Oncopeltus* accumulated its enemy fauna over the course of evolutionary time could be linked to the inhibition assays of predator target sites to understand the order of assembly of its defence arsenal [67,68].

The differential effect of cardenolides on the different predator enzymes supports the idea that natural enemies can foster defence diversification and that the assembly of sequestered defensives might depend on which selection pressure predators impose [51,68]. We did not test predator responses to isolated chemical defences (e.g. [26]). In the wood tiger moth, *Arctia plantaginis*, the defensive neck fluids are a more effective defence against bird predators than against invertebrates, and abdominal fluids provide greater defence against invertebrates than against birds [51]. We suggest that tests of single compounds and defensive mixture will be useful for understanding how toxicity relates to predator deterrence [69,70] and whether compounds act synergistically, additively or antagonistically [53,71]. We did not test non-sequestered cardenolides on predator enzymes because we were unable to isolate them in sufficient concentration or purity [40]. Testing the effects of the non-sequestered compounds on predators and the bug’s target sites would also provide more information on the costs or other constraints on sequestration (e.g. [27]). For example, certain cardenolides can be detrimental to insect growth [72], as well as redox state [56]. Sequestration might also be constrained by transport and modification of specific compounds [48,73]. Testing the uptake, transport, modification and accumulation of the cardenolides that we found in similar concentrations in the body and defensive fluid (i.e. 16α-hydroxycalotropin), and comparing to those that were more abundant in the bugs than the seeds (frugoside and gofruside; [40]), would be useful for establishing which key metabolic process the bugs use when feeding on *Asclepias* (refer to Agrawal *et al.* [48]).

## 5. Conclusion

The results of the present study suggest that the sequestration of cardenolides by *O. fasciatus* is shaped by the forces of predation pressure. While cardenolides have long been known for their role in shaping predator–prey interactions, this is among the first tests *in vitro* of specific cardenolides sequestered by milkweed herbivores on the target site of a range of predators (see also [27]). Tests of whether chemical variation in large milkweed bugs correlates with measured predation pressure will be important for understanding if natural selection drives investment in chemical defences in this species.

## Data Availability

The data and analysis pipeline are available in the Max Planck open-access data repository Edmond [45]. Methods and supplementary analyses are available in the supplementary material [74].
